# Infection Control Programs and Antibiotic Control Programs to Limit Transmission of Multi-Drug Resistant *Acinetobacter baumannii* Infections: Evolution of Old Problems and New Challenges for Institutes

**DOI:** 10.3390/ijerph120808871

**Published:** 2015-07-30

**Authors:** Chang-Hua Chen, Li-Chen Lin, Yu-Jun Chang, Yu-Min Chen, Chin-Yen Chang, Chieh-Chen Huang

**Affiliations:** 1 The Infectious Disease Research Center, 135 Nan-Hsiao Street, Changhua 500, Taiwan; E-Mail: 3344@cch.org.tw; 2 Division of Infectious Disease, Department of Internal Medicine, 135 Nan-Hsiao Street, Changhua 500, Taiwan; E-Mail: 106018@cch.org.tw; 3 Epidemiology and Biostatics Center, Changhua Christian Hospital, 135 Nan-Hsiao Street, Changhua 500, Taiwan; E-Mail: 83686@cch.org.tw; 4 Department of Pharmacy, Changhua Christian Hospital, 135 Nan-Hsiao Street, Changhua 500, Taiwan; E-Mail: 30855@cch.org.tw; 5 Department of Life Science, National Chung Hsing University, 250 Kuo-Kuang Road, Taichung 402, Taiwan; E-Mail: cchuang@dragon.nchu.edu.tw

**Keywords:** infection control programs, antibiotic control programs, *Acinetobacter baumannii* complex, multidrug-resistant

## Abstract

Background: *Acinetobacter baumannii* complex (*A. baumannii*) has been isolated worldwide. The rapid spread of multidrug-resistant *A. baumannii* complex (MDRAB) in clinical settings has made choosing an appropriate antibiotic to treat these infections and executing contact precautions difficult for clinicians. Although controlling the transmission of MDRAB is a high priority for institutions, there is little information about MDRAB control. Therefore, this study evaluated infection control measures for *A. baumannii* infections, clusters and outbreaks in the literature. Methods: We performed a review of OVID Medline (from 1980 to 2015), and analyzed the literature. Results: We propose that both infection control programs and antibiotic control programs are essential for control of MDRAB. The first, effective control of MDRAB infections, requires compliance with a series of infection control methods including strict environmental cleaning, effective sterilization of reusable medical equipment, concentration on proper hand hygiene practices, and use of contact precautions, together with appropriate administrative guidance. The second strategy, effective antibiotic control programs to decrease *A. baumannii*, is also of paramount importance. Conclusion: We believe that both infection control programs and antibiotics stewardship programs are essential for control of MDRAB infections.

## 1. Introduction

*Acinetobacter baumannii* complex (*A. baumannii*) has been noticed worldwide, and the rapid spread of multidrug-resistant *A. baumannii* complex (MDRAB) in clinical institutions has made choosing an adequate antibiotic to treat these infections and executing contact precaution to isolate these MDRAB difficult for clinicians. The reported incidence of *A. baumannii* infections has substantially increased during the past decades [1], and Nhu’s work highlights the emergence of a carbapenem-resistant *A. baumannii* complex (CRAB) and a rising incidence of CRAB in the intensive care unit (ICU) [2]. Intra-hospital and inter-hospital spread of CRAB and colistin-resistant *A. baumannii* complex have appeared in ICU [3,4], and institutional outbreaks caused by MDRAB are a growing public-health problem.

Important changes with regard to *A. baumannii* infections were noted in Villar’s study [5]. An increase in admissions of affected patients to conventional wards and an increase in infection patterns for non-nosocomial health care-associated infections (HAIs) are noted. In addition, epidemic clones of MDRAB seem to combine with antimicrobial resistance, the ability to spread, and clinical virulence [5]. According to data from the Taiwan Nosocomial Infection Surveillance System (TNIS), there was an increase in the proportion of number of HAIs caused by CRAB over that by all *A. baumannii* [6]. Meanwhile, CRAB increased in the ICUs, although the overall rate of HAIs has decreased. This longitudinal multicenter surveillance program showed significant increase and nationwide emergence of extensively drug-resistant *A. baumannii* complex in Taiwan over the years from the Taiwan Surveillance of Antimicrobial Resistance (TSAR) [7]. In addition, colistin-resistant *A. baumannii* occurred almost exclusively among patients who had received colistin methansulfonate for treatment of carbapenem-resistant and colistin-susceptible *A. baumannii* infections [3,8].

The current problems are that *A. baumannii* is still a major pathogen in institutes worldwide and physicians realize that both infection control and antibiotics stewardship are very essential to control *A. baumannii* infections [9]. Treatment of MDRAB infections is a great challenge for physicians as is control of MDRAB spread. Although controlling the transmission of MDRAB is a high priority for institutes, there is little information about detailed infection control measures and antibiotics stewardship steps to control MDRAB [3,8].

Therefore, the purpose of our study is to review the literature in order to evaluate and prioritize risk factors and infection control measures for *A. baumannii* infections, clusters, and outbreaks.

## 2. Materials and Methods

### 2.1. Definition of A. baumannii in this Study

We traced back more than 30 genomic species which have been identified within genus Acinetobacter, 17 of which have been assigned valid names [10]. *A. baumannii* (gen sp 2) and Acinetobacter gen sp 3 and Acinetobacter gen 13 TU are pathogenic in human beings [11,12,13]. Because these three species are closely related genetically, and cannot be accurately differentiated by routine phenotypic methods [14], the term *A. baumannii* has been used to refer to all three in this study.

### 2.2. Data Sources and Searches

We performed systematic searches of the literature in the following bibliographical databases: MEDLINE (PubMed), CINAHL, EMBASE and the ISI Web of Sciences. Search criteria included articles published in the period from January 1980 to March 2015, and only included articles published in English, Spanish, French, German and Chinese. The search strategy included the Medical Subject Headings (MeSH) and text words “*Acinetobacter baumannii*”, “prevention and control”, “antibiotic and resistant”, “infection control program”, “antibiotic control program” and “multi-drug resistant *Acinetobacter baumannii*”, and as limited to studies conducted after 1980.

### 2.3. Study Selection

Two investigators (CCH and CYM) independently performed the literature search and the study selection. Any disagreement was resolved by a third author (CYC), and a final consensus was reached among all authors. Studies were considered eligible if they reported a strategy to prevent MDRAB infections. The studies that did not report any strategies or effectiveness studies were excluded. From 72,429 papers we extracted 154 papers, all of which were reviewed. We summarized 72 papers to aggregate the risk factors for acquisition of *A. baumannii* infections, and we summarized 62 papers to compile the infection control measures of *A. baumannii* infections.

## 3. Results

Management of MDRAB infections is a great challenge for physicians. Physicians have realized that both infection control and antibiotics stewardship are very essential to control *A. baumannii* infections [15,16].

**Figure 1:**
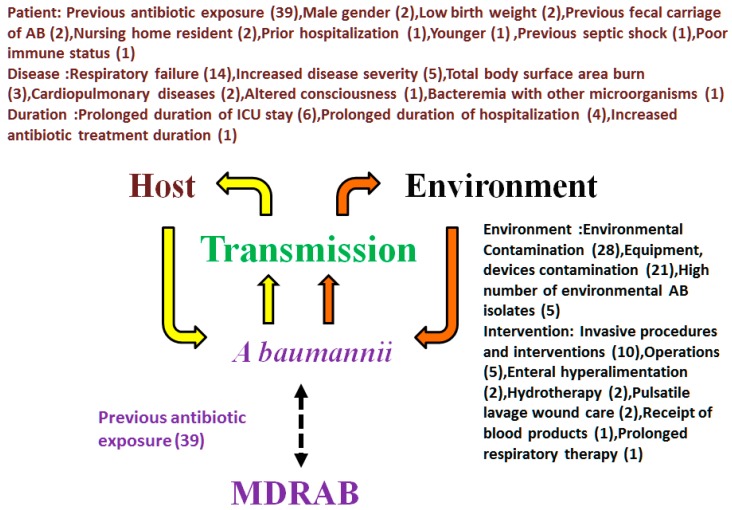
Risk factors for acquisition of *A. baumannii* infections (including acquisition of multi-drug-resistant *A. baumannii*). The acquisition of *A. baumannii* infections include three factors, which are source, host, and transmission. The reference number of those reported risk factors is marked between open parenthesis and close parenthesis; carbepenem resistant *A. baumannii* (CRAB); MDRAB: multidrug-resistant *A. baumannii*.

**Figure 2:**
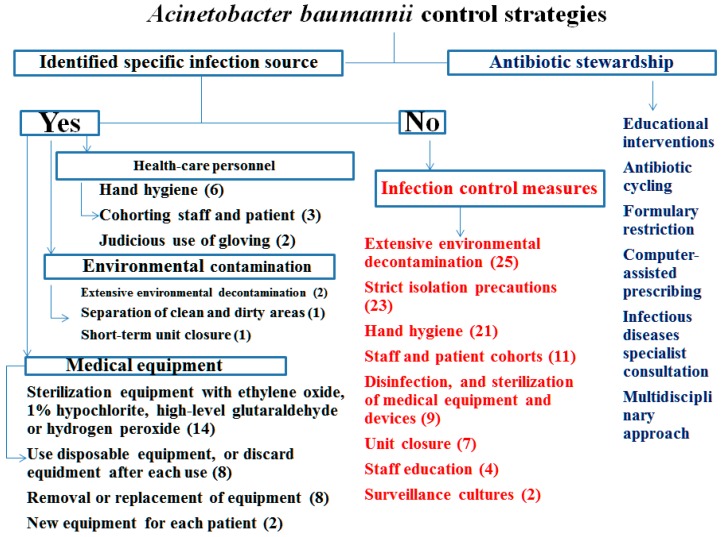
Summary of infection control measures directed against *A. baumannii* outbreak and cluster; the infection control measures for *A. baumannii* outbreak and cluster are summarized. The literature number of those reported risk factors is marked between open parenthesis and close parentheses.

Figure 1 and Supplementary Table S1 showed specific characteristics of affected patients who got *A. baumannii* and MDRAB infections. Apart from the routine general control measures recommended for institutional outbreaks caused by multidrug-resistant organisms [17], both “identified source” and “no identified source” of transmission of MDRAB are summarized in Supplementary Table S2. Figure 2 shows the concept frame for the implementation of specific control measures against *A. baumannii* infections.

Particular attention should be paid to effective environmental cleaning (28), administration of broad-spectrum antimicrobial agents (39), and respiratory failure with mechanical ventilation (14), invasive procedures (10), and longer duration of ICU stay (6).

Infection control measures need to be implemented for a long time at every institute. Restricting excessive antimicrobial agents is important, but it is a dilemma to decide the best way to treat patients and control resistant *A. baumannii.* Although it is an old problem, *A. baumannii* infections have become an increasing challenge nowadays.

## 4. Discussion

It is obvious that organizing a multi-disciplinary unit to control the transmission of *A. baumannii* infections is necessary. In order to stop the transmission of *A. baumannii* infections, blocking the transmission chain (Figure 1) and following the infection control measures (Figure 2) are very important, but it is a dilemma to decide which is the best way to go*.* This current challenge has motivated new attempts at early detection, effective control and eventual prevention of *A. baumannii* HAIs.

The transmission chain includes two important components to control the transmission of *A. baumannii* infections. Source control including effective environmental cleaning, adequate administration of broad-spectrum antimicrobial agents, restriction usage of mechanical ventilation for respiratory failure, and avoidance of longer duration in the ICU are most essential. In addition, host factor control is also essential, but it is difficult to control those host factors in clinical practice.

Specific demographic characteristics of affected patients include advanced age, immune suppression status, extended hospital stay, and previous administration of antibiotics [18,19,20,21]. Risk factors also include higher number of interventions [22,23,24,25,26,27,28], including receipt of transfusions of blood products [29], usage of enteral hyperalimentation [30,31], pressure transducers [31], and hydrotherapy [29,32]; prolonged respiratory therapy [33]; and operation [34,35,36,37], as well as host underlying conditions [23,24,32,34,36,38,39,40,41,42,43,44] and nursing home stay [28,43]. We believe there is significant room for improvement in how these host factors are managed in decreasing disabilities and promoting healthy lifestyles. We think managing the host factors has the potential to improve the clinical outcome for prevention of *A. baumannii* transmission.

Particular attention should be paid to effective environmental cleaning [29,45,46,47,48,49,50,51,52,53]. Disinfectants should be applied for an adequate period of time to achieve sterilization. Disinfection of potentially contaminated medical equipment should be done meticulously [50,53,54]. Special attention is required for the sterilization of mechanical ventilators [53,55,56,57,58] Use of closed suction systems can also prevent environmental contamination with *A baumannii* [45,50]*.* We believe most of those infection control measures have to be rigorously enacted to improve the clinical outcomes for prevention of *A. baumannii* transmission.

Our experience presents an evaluation of the long-term effectiveness of infection control programs (including hand hygiene champions, antibiotics control program and process of cluster/outbreak, Supplementary Table S3 and Figure S1) that describes effects in the control of CRAB over a contemporary nine-year period according to international accreditation programs (that is, Joint Commission International accreditation) and national accreditation programs (Hospital Accreditation and Healthcare-Related Infection Control Audit and Quality Improvement by Taiwan Joint Commission on Hospital Accreditation). Much evidence that shows that accreditation programs improve the process of patient care provided by healthcare services [59,60]. Our experience also provides another piece evidence for improvement of infection control and prevention.

We acknowledge several limitations to our recommendations. First, administrations of institutes sometimes lack the strong commitment required to support the principles and policies of infection control because it takes time to educate staff and to wait for positive changes to yield clinical benefits. Administrative commitment and measures for organizing an effective team and providing timely feedback of information regarding *A. baumannii* transmission are fundamental [61,62]. Education of hospital staff on a regular basis and frequent revision of the control measures used are also essential [44,47,62,63,64,65]. It should also be mentioned that infection control measures might need to be implemented for a long time before control of an outbreak is achieved [66]. Second, each institute has its principles and polices for infection control, but the compliance and adherence of health-care workers (HCWs) regarding those elements is the essential key to achieving an excellent outcome. Adherence of HCWs to hand-hygiene protocols is of supreme importance for the restraint of *A. baumannii* transmission [29,45,46,53,67,68], and adherence of HCWs as a precaution [46,53], will also contribute in the control of *A. baumannii* outbreaks. Third, because the misuse and overuse of antibiotics is also a common problem in Taiwan, we emphasized the importance of antibiotic stewardship in the improvement of the trend of *A. baumannii* transmission. Effective antibiotics control policy is difficult to implement at each institute. Fourth, many typing methods for *A. baumannii* epidemiological studies from phenotypic to molecular methods to detect outbreak of *A*. *baumannii* have been proposed [69]. In this study, each method has its own advantages and disadvantages, and the selection of an appropriate genotyping method has not been reviewed because most situations depend on clinical objectives and conditions.

There are some limitations of this study. First, this is not an evidence-based methodology and not a systemic review because most of the articles reviewed are not of double-blinded, case-control studies, and most of them do not assess methodological quality and homogeneity. Second, our recommendations according to the study selection of literature review do not allow for precise appropriate risk assessment methods and efficient control measures because most clinical situations depend on specific objectives and local conditions.

## 5. Conclusions

In order to control *A. baumannii* infections, these strategies are very critical. Ameliorating these risk factors is essential, but it is not easy to execute. Restricting the prescription of antimicrobial agents is important, but it is difficult to decide which regimen is the best way to treat patients during empirical therapy*. A. baumannii* infections is an old problem, but *A. baumannii* infections have become a growing challenge nowadays. We reviewed the literature to summarize the advanced findings for control of MDRAB infections. In the prevention of MDRAB infections, it should be mentioned that infection control measures might need to be implemented for a long time before control of an outbreak is achieved. In some cases, this might require the closure of hospital units. It is also noteworthy that the control of outbreaks involving long-term care facilities may be more demanding than nosocomial ones, because of the relative shortage of appropriate resources. To reduce the transmission of *A. baumannii* infections and decrease the emergence of resistance in MDRAB, it is essential to advance rational prescription of antimicrobials with support from clinical microbiologists in institutions. Dedicated prescription of carbapenems should be one of several important actions to control the increase of CRAB. Hand hygiene and contact precaution are also essential to suppress the spread of infection. Surveillance of antibiotic susceptibility tests is essential in providing useful information for physicians to choose adequate antibiotics at empirical periods.

## Supplementary Files

Supplementary File 1
