# Supplementary material for: Infection Control Programs and Antibiotic Control Programs to Limit Transmission of Multi-Drug Resistant Acinetobacter baumannii Infections: Evolution of Old Problems and New Challenges for Institutes
Source: Int J Environ Res Public Health. 2015 Jul 30;12(8):8871–82. doi: 10.3390/ijerph120808871 (PMC4555253; doi:10.3390/ijerph120808871)
Supplement: Supplementary File 1 [file ijerph-12-08871-s001.pdf]

*Supplementary Information*

# Infection Control Programs and Antibiotic Control Programs to Limit Transmission of Multi-Drug Resistant *Acinetobacter baumannii* Infections: Evolution of Old Problems and New Challenges for Institutes

**Table S1.** Risk factors for acquisition of AB infection (including acquisition of multi-drug-resistant *Acinetobacter baumannii).*

| Author/Year | Study Design | Risk Factors |
|---|---|---|
| (1) Environment | | |
| environmental contamination | | |
| Ho CM/2013 | Case-control study, prospective | Environment contamination |
| Hosoglu S/2013 | Case-control study | Environment contamination |
| Pimentel JD/2005 | Non-case-control study | Environmental contamination (improper cleaning methods used on respiratory equipment) |
| Wroblewska MM/2004 | Non-case-control study | Environmental contamination (suctioning equipment of the neurosurgery ward, mattress, resuscitation equipment, steel trolley) |
| El Shafie SS/2004 | Non-case-control study | Environmental contamination (bedrails, curtains, suction with vacuum, ambu bags, ventilation filters) |
| Ferreira AC/2004 | Non-case-control study | Cross-transmission |
| Mittal N/2003 | Non-case-control study | Environmental contamination (intravenous catheters, washbasin) |
| Wang SH/2003 | Non-case-control study | Environmental contamination (bed rails, bedside tables, surface of ventilators and infusion pumps, water for nasogastric feeding and ventilator rinsing, and sinks) |
| Melamed R/2003 | Non-case-control study | Environmental contamination (hygroscopic bandages and umbilical catheters) |

**Table S1.** *Cont.*

| Author/ Year | Study Design | Risk Factors |
|---|---|---|
| Hsueh PR/2002 | Non-case-control study | Environmental contamination (ventilator monitor board and tips of feeding syringe) |
| Huang YC/2002 | Non-case-control study | Environmental contamination (contaminated hands of healthcare workers, bed rails, ventilator, incubator, heater) |
| Aygun G/2002 | Non-case-control study | Environmental contamination (beds, tables, infusion pumps, pulse oximeters, blood pressure cuffs, dresser, cupboard, service desk) |
| Levidiotou S/2002 | Non-case-control study | Environmental contamination (trunking) |
| Das I/2002 | Non-case-control study | Environmental contamination (bed surfaces, surfaces of equipment, dried floor mops and curtains) |
| Jeena P/2001 | Non-case-control study | Environmental contamination (ventilator dials, table surfaces, catheter suction tubing and telephones in the duty room) |
| Roberts SA/2001 | Non-case-control study | Environmental contamination (door handle, ventilator control device, support stands and hands of healthcare workers) |
| Pillay T/1999 | Non-case-control study | Environmental contamination (surface of a laryngoscope, suction bottles and catheters) |
| Cox MT/1998 | Non-case-control study | Environmental contamination (sinks) |
| Go ES/1994 | Non-case-control study | Environmental contamination (beds, tables, intravenous drip supports, ventilators and personnel hands) |
| Tankovic J/1994 | Non-case-control study | Environmental contamination (sinks, furniture, gowns, windowsills) |
| Ahmed J/1994 | Non-case-control study | Environmental contamination (peak flow meter) |
| Cefai C/1990 | Non-case-control study | Environmental contamination (ventilator tubing) |
| Allen KD/1987 | Non-case-control study | Environmental contamination (bed linen, curtains, mattresses, lamp, hands of healthcare workers) |
| Stone JW/1986 | Non-case-control study | Environmental contamination (“Ambu” resuscitation device) |
| Number of environmental AB isolates | | |
| Marchaim D/2007 | Retrospective analysis of prospectively collected data | In 65% of cases, quantification of transmission opportunities (TOPs) involving patients with the same clone is greater than the number of TOPs involving patients with different clones ($p = 0.01$). |
| Denton M/2004 | Non-case-control study | Number of environmental AB isolates obtained during each monthly screening ($p = 0.004$) |
| Corbella X/2000 | Case-control study | Admission into a ward with a high density of CR AB (RR 1.73; 95% CI 1.21–2.47) |
| D'Agata EM/2000 | Case-control study, matched | Exposure to a significantly higher number of patients with MDR AB infections (OR 1.1; 95% CI 1.01–1.2; $p = 0.02$) |
| Mulin B/1995 | Case-control study, prospective | Previous stay in a unit (OR 9.1; 95% CI 1.31–63.20) |

**Table S1.** *Cont.*

| Author/ Year | Study Design | Risk Factors |
|---|---|---|
| Previous faecal carriage | | |
| Corbella X/2000 | Case-control study | Previous fecal carriage of CR AB (RR 35.3; 95% CI 7.2–173.1) |
| Wisplinghoff H/1999 | Case-control study, retrospective | Prior nosocomial colonization with AB at a distant site (OR 26.23; 95% CI 4.68–147) |
| (2) Antibiotic exposure | | |
| previous exposure to antibacterial agent | | |
| Zhang HM/2014 | Non-case-control study | Previous antibacterial use |
| Ma MY/2013 | Non-case-control study | Usage of broad-spectrum antibiotics (OR = 3.054, 95% CI 1.009–6.550, $p = 0.004$). |
| Jung JY/2010 | Case-control study, retrospective | Prior antimicrobial therapy |
| Romanelli RM/2009 | Case-control study | Carbapenem use showed a tendency towards a statistical association ($p = 0.07$) |
| Romanelli RM/2009 | Case-control study | Third-generation cephalosporin use ($p = 0.09$ ) |
| Young LS/2007 | Non-case-control study | Receipt of fluconazole(OR, 73.3; $p < 0.01$), |
| Young LS/2007 | Non-case-control study | Receipt of levofloxacin(OR, 11.5; $p = 0.02$) |
| von Dolinger de Brito D/2006 | Non-case-control study | Exposure to antibiotics |
| Garnacho-Monter J/2006 | Case-control study, retrospective | Prior exposure to imipenem (OR 4; 95% CI 1.1–29.8) |
| del Mar Tomas M/2005 | Case-control study | Previous treatment with third-generation cephalosporins (OR 10; 95% CI 1.1–92.2) |
| Carbonne A/2005 | Case-control study | Previous treatment with third-generation cephalosporins (OR 10; 95% CI 1.1–92.2) |
| Abbo A/2005 | Case-control study, matched | Prior treatment with antimicrobial drugs (particularly metronidazole) (OR 4.74; 95% CI 1.31–17.15) |
| Nseir S/2005 | Case-control study, prospective | Fluoroquinolone use (OR 3.3; 95% CI 1.7–6.5) |
| Lee SO/2004 | Case-control study | Prior exposure to imipenem (OR 9.18; 95% CI 3.99–21.13) |
| Lee SO/2004 | Case-control study | Prior exposure to third-generation cephalosporins (OR 2.11; 95% CI 1.13–3.95) |
| Smolyakov R/2003 | Case-control study | Prior aminoglycoside therapy ($p = 0.003$) |
| Mahgoub S/2002 | Case-control study, retrospective | Prior antibiotic use ($p = 0.035$) |
| Hsueh PR/2002 | Non-case-control study | Use of carbapenems |
| Hsueh PR/2002 | Non-case-control study | Use of ciprofloxacin |

**Table S1.** *Cont.*

| Author/Year | Study Design | Risk Factors |
|---|---|---|
| Chen CH/2002 | Case-control study, retrospective | Administration of broad-spectrum antimicrobial agents ($p < 0.05$) |
| Landman D/2002 | Non-case-control study | Cephalosporin use at each hospital ($p = 0.004$) |
| Jeena P/2001 | Non-case-control study | Prior use of broad-spectrum antimicrobial agents |
| Manikal VM/2001 | Non-case-control study | Use of third-generation cephalosporins ($p = 0.03$) |
| D'Agata EM/2000 | Case-control study, retrospective | Administration of broad-spectrum antimicrobial agents |
| Husni RN/1999 | Case-control study, retrospective | Prior use of ceftazidime ($p < 0.01$) |
| Spanik S/1999 | Case-control study | Previous prophylaxis with ofloxacin ($p < 0.05$) |
| Spanik S/1999 | Case-control study | Prior therapy with first-generation($p < 0.05$) |
| Spanik S/1999 | Case-control study | Prior therapy with second-generation ($p < 0.05$) |
| Spanik S/1999 | Case-control study | Prior therapy with third-generation cephalosporins ($p < 0.01$) |
| Spanik S/1999 | Case-control study | Prior use of ceftazidime (OR 6; 95% CI 1.1–35) |
| Spanik S/1999 | Case-control study | Prior therapy with aminoglycosides ($p < 0.01$) |
| Spanik S/1999 | Case-control study | Prior therapy with imipenem ($p < 0.001$) |
| Koeleman JG/1997 | Case-control study | Treatment with broad-spectrum antibiotics (OR 4.3; 95% CI 1.0–18.1) |
| Levin AS/1996 | Case-control study | Number of antecedent antibacterials ($p = 0.025$) |
| Scerpella EG/1995 | Case-control study | Use of third-generation cephalosporin ($p < 0.05$) |
| Peacock Jr JE/1981 | Case-control study | Prior therapy with cephalosporins ($p = 0.018$) |
| Peacock Jr JE/1981 | Case-control study | Prior therapy with aminoglycosides ($p = 0.08$) |
| Go ES/1994 | Non-case-control study | Use of imipenem |
| Struelens M/1993 | Case-control study, retrospective | Administration of broad-spectrum antimicrobial agents |

**Table S1.** *Cont.*

| Author/Year | Study Design | Risk Factors |
|---|---|---|
| (3) Host | | |
| Longer duration of antibiotic treatment | | |
| Nseir S/2005 | Case-control study, prospective | Longer duration of antibiotic treatment (OR 1.1; 95% CI 1.0–1.2/day) |
| imipenem as monotherapy | | |
| del Mar Tomas M/2005 | Case-control study | Administration of imipenem as monotherapy (OR 11.12; 95% CI 2.33–53.09) |
| mechanical ventilation , and respiratory failure | | |
| Ma MY/2013 | Non-case-control study | Mechanical ventilation (odds ratio (OR) = 2.957, 95%confidence interval (95% CI) 1.106–6.253, $p = 0.023$) |
| Jung JY/2010 | Case-control study, prospective | Respiratory failure at the time of ICU admission |
| Jung JY/2010 | Case-control study, prospective | Maintenance of mechanical ventilation |
| Romanelli RM/2009 | Case-control study | Mechanical ventilation use ($p = 0.003$) |
| Young LS/2007 | Non-case-control study | Duration of intubation(OR, 1.4 per day intubated; $p < 0.01$) |
| Abbo A/2005 | Case-control study, matched | Mechanical ventilation (OR 6.27; 95% CI 2.27–17.33) |
| Maslow JN/2005 | Case-control study, prospective | Mechanical ventilation ($p < 0.001$) |
| Simor AE/2002 | Case-control study | Mechanical ventilation (OR 1.1/day; 95% CI 1.0–1.1) |
| Mahgoub S/2002 | Case-control study, retrospective | Mechanical ventilation ($p < 0.001$) |
| Corbella X/2000 | Case-control study | Previous therapy with carbapenems (RR 4.58; 95% CI 1.34–15.6) |
| Cox MT/1998 | Non-case-control study | Mechanical ventilation ($p < 0.05$) |
| Baraibar J/1997 | Case-control study, prospective | ARDS (OR 9.73; 95% CI 1.60–59.24) |
| Levin AS/1996 | Case-control study | Intubation ($p = 0.049$) |
| Struelens M/1993 | Case-control study, retrospective | Mechanical ventilation |
| Beck-Sague CM/1990 | Case-control study | Mechanical ventilation ($p = 0.0113$) |

**Table S1.** *Cont.*

| **Author/Year** | **Study Design** | **Risk Factors** |
|---|---|---|
| Pulmonary aspiration event | | |
| Jung JY/2010 | Case-control study, prospective | Maintenance of endotracheal tube instead of switching to a tracheostomy |
| Baraibar J/1997 | Case-control study, prospective | Pulmonary aspiration (OR 2.90; 95% CI 0.80–10.53) |
| duration of ICU stay | | |
| Ma MY/2013 | Non-case-control study | ICU stay time ≥ 7 days (OR = 2.991, 95% CI 1.135–6.544, $p = 0.022$) |
| Maslow JN/2005 | Case-control study, prospective | ICU admission ($p < 0.001$) |
| Lee SO/2004 | Case-control study | ICU stay (OR 21.54; 95% CI 10.73–43.23) |
| Smolyakov R/2003 | Case-control study | ICU admission ($p < 0.0001$) |
| Koeleman JG/1997 | Case-control study | Prior ICU stay ($p = 0.002$) |
| Mulin B/1995 | Case-control study, prospective | Longer ICU stay before colonization (OR 1.23; 95% CI 1.12–1.35) |
| Scerpella EG/1995 | Case-control study | Longer duration of ICU stays ($p < 0.05$) |
| Peacock Jr JE/1981 | Case-control study | Length of ICU stay ($p = 0.002$) |
| duration of hospitalization | | |
| von Dolinger de Brito D/2008 | Non-case-control study | Duration of hospitalization > or = 7 days |
| Maslow JN/2005 | Case-control study, prospective | Total hospital days ($p < 0.001$) |
| Koeleman JG/1997 | Case-control study | Length of stay >7 days (OR 5.3; 95% CI 1.3–22.6) |
| Scerpella EG/1995 | Case-control study | longer duration of hospital stays ($p < 0.05$) |
| Prior hospitalization | | |
| Mahgoub S/2002 | Case-control study, retrospective | Prior hospitalization ($p = 0.014$) |
| invasive procedures , intervention | | |
| Xia Y/2012 | Case-control study | exposure to bronchoscopy (OR, 22.5; 95% CI, 2.07–244.84; $p = 0.005$) |
| Jung JY/2010 | Case-control study, prospective | recent central venous catheter insertion |
| Romanelli RM/2009 | Case-control study | central venous catheter use ($p = 0.04$). |

**Table S1.** *Cont.*

| **Author/Year** | **Study Design** | **Risk Factors** |
|---|---|---|
| Young LS/2007 | Non-case-control study | exposure to bronchoscopy ( OR, 22.7; $p = 0.03$), |
| von Dolinger de Brito D/2006 | Non-case-control study | exposure to invasive devices |
| del Mar Tomas M/2005 | Case-control study | Arterial catheter (OR 1.13; 95% CI 1.03–1.25) |
| Maslow JN/2005 | Case-control study, prospective | Central venous catheterization ($p < 0.001$) |
| Wong H/2002 | Case-control study, retrospective | Number of intravascular lines placed ($p < 0.05$) |
| Mahgoub S/2002 | Case-control study, retrospective | Foley catheter ($p < 0.001$) |
| Mahgoub S/2002 | Case-control study, retrospective | Tracheostomy ($p = 0.001$) |
| Receipt of blood products | | |
| Simor AE/2002 | Case-control study | Receipt of blood products (OR 10.8; 95% CI 3.4–34.4) |
| Hyperalimentation | | |
| Mulin B/1995 | Case-control study, prospective | Enteral hyperalimentation (OR 7.47; 95% CI 1.36–40.95) |
| Beck-Sague CM/1990 | Case-control study | Hyperalimentation ($p = 0.018$) |
| pressure transducers | | |
| Beck-Sague CM/1990 | Case-control study | Duration of transducer use at an insertion site (in days) ($p = 0.0064$) |
| hydrotherapy | | |
| Simor AE/2002 | Case-control study | Procedures performed in the hydrotherapy room (OR 4.1; 95% CI 1.3–13.1) |
| Wisplinghoff H/1999 | Case-control study, retrospective | Use of hydrotherapy (OR 5.5; 95% CI 1.11–27.76) |
| respiratory therapy | | |
| Peacock Jr JE/1981 | Case-control study | Prolonged respiratory therapy ($p = 0.006$) |
| Operation | | |
| Katsaragakis S/2005 | Case-control study, prospective | reoperation ($p = 0.01$; OR, 8.45; 95% CI, 1.52–46.85) |
| Borgmann S/2004 | Non-case-control study | Surgery in a specific operation theatre where contaminated surgery class IV (infected, dirty) was performed |

**Table S1.** *Cont.*

| **Author/Year** | **Study Design** | **Risk Factors** |
|---|---|---|
| Koeleman JG/1997 | Case-control study | Surgery ($p$ = 0.017) |
| Baraibar J/1997 | Case-control study, prospective | Neurosurgery (OR 10.03; 95% CI 1.55–64.90) |
| Baraibar J/1997 | Case-control study, prospective | Head trauma (OR 5.17; 95% CI 0.88–30.34) |
| Gender | | |
| Abbo A/2005 | Case-control study, matched | Male sex (OR 3.84; 95% CI 1.63–8.99) |
| Wisplinghoff H/1999 | Case-control study, retrospective | Female sex (OR 5.13; 95% CI 1.20–21.83) |
| Mulin D/1995 | Case-control study, prospective | Male sex (OR 2.95; 95% CI 1.00–8.96) |
| age | | |
| Lee SO/2004 | Case-control study | Age (OR 1.03; 95% CI 1.01–1.05) |
| Melamed/2003 | Non-case-control study | Younger age |
| Immune status | | |
| Zhang HM/2014 | | non-case-control study |
| bacteremia with other than *A. baumannii* micro-organism | | |
| Jung JY/2010 | Case-control study, prospective | Bacteremia caused by other microorganism after colonization by MDR AB |
| previous septic shock | | |
| Katsaragakis S/2005 | Case-control study, prospective | Previous septic shock ($p$ = 0.04; odds ratio (OR), 9.83; 95% confidence interval (CI), 1.003–96.29) |
| Low birth weight | | |
| von Dolinger de Brito D/2007 | Non-case-control study | Birth weight <1500 g, age < or = 7 days |
| Melamed R/2003 | Non-case-control study | Lower birthweights |
| Conscious change | | |
| Ma MY/2013 | Non-case-control study | Coma (GCS < 8,OR = 2.894, 95% CI 1.803–7.462, $p$ = 0.010) |

**Table S1.** *Cont.*

| Author/Year | Study Design | Risk Factors |
|---|---|---|
| Cardiopulmonary disease | | |
| Young LS/2007 | Non-case-control study | Presence of chronic pulmonary disease (OR, 77.7; $p = 0.02$) |
| Abbo A/2005 | Case-control study, matched | Cardiovascular disease (OR 3.35; 95% CI 1.44–7.77) |
| wound care | | |
| Maragakis LL/2004 | Case-control study | Pulsatile lavage wound care (OR 36; 95% CI 2.8–1721) |
| Koeleman JG/1997 | Case-control study | Wounds ($p = 0.044$) |
| Nursing home | | |
| Maragakis LL/2004 | Case-control study | Nursing home residence (OR 36; 95% CI 2.2–1833) |
| Mahgoub S/2002 | Case-control study, retrospective | Nursing home residency ($p = 0.001$) |
| Time from admission to positive-culture date | | |
| Lee SO/2004 | Case-control study | Time from admission to positive-culture date (OR 1.02; 95% CI 1.002–1.03) |
| Burn | | |
| Wisplinghoff H/1999 | Case-control study, retrospective | Total body surface area burn of >50% (OR 6.13; 95% CI 1.21–37.80) |
| Presence of infection at admission | | |
| Romanelli RM/2009 | Case-control study | Prior infection ($p = 0.002$) |
| Lortholary O/1995 | Case-control study, prospective | Presence of infection at admission ($p < 0.001$) |
| greater disease severity | | |
| Young LS/2007 | Non-case-control study | Higher Acute Physiology and Chronic Health Evaluation II score (odds ratio (OR), 1.1 per point increase; $p = 0.06$), |
| Brahmi N/2007 | Case-control study, retrospective | Simplified Acute Physiological Score (SAPS II) |
| Wong H/2002 | Case-control study, retrospective | APACHE II score on admission ($p < 0.05$) |
| Fierobe L/2001 | Case-control study | High individual therapeutic intervention score (OR 2.67; 95% CI 0.99–7.22) |
| Lortholary O/1995 | Case-control study, prospective | APACHE II score ($p < 0.05$) |

Abbreviation: TOPs = quantification of transmission opportunities Study; SAPSII = Simplified Acute Physiological Score; OR = odds ratio; CI = confidence interval.

**Table S2.** Summary of infection control measures directed against the *A. baumannii* outbreak and cluster.

| Authors/Year | Control Measures Directed Against the Source | Source/Predominant Involved Sites |
|---|---|---|
| Outbreak (or Clusters) with an identified common source | | |
| Alfandari S/2014 | Strict isolation precautions, staff and patient cohorting, and extensive environmental decontamination | blood pressure cuffs Velcro/NM |
| Xia Y/2012 | Appropriate bronchofiberscope reprocessing and environmental decontamination | Bronchofiberoscopy/NM |
| Wroblewska MM/2004 | NM | suctioning equipment/CSF |
| Melamed R/2003 | Discontinuing the use of the bandages | hygroscopic bandages/soft tissue |
| Kappstein I/2000 | Water taps and aerators removed and replaced, reinforcement of hand antisepsis and judicious use of gloving | Water taps in staff room with mesh aerators/Blood |
| Pillay T/1999 | New suction catheter for each neonate, short-term unit closure, cohorting staff, hand hygiene | Suction catheter and bottle/NM |
| Yoo JH/1999 | Replacement of ventilators | "Y" piece of ventilator/NM |
| Bellamore E/1997 | New acetylcysteine nebulizer for each patient, enforcement of proper handling of multidose vials | Multidose acetyl- cysteine nebulization to multiple patients (presumed)/NM |
| Snelling AM/1996 | Sterilization of probes with $H_2O_2$ free radicals | Temperature probe of ventilator humidifier/NM |
| Kaul R/1996 | Ethylene oxide terminal sterilization of temperature probes, hand washing, cohorting culture-positive patients, separation of clean and dirty areas in respiratory therapy department | Ventilator temperature probes/NM |
| Weernink A/1995 | Elimination of feather pillows, switch to synthetic pillows, washing pillows at 85 °C | Feather pillows washed at low temperature/Soft tissue |
| Ahmed J/1994 | High-level glutaraldehyde disinfection of flow meter, use of disposable mouthpiece, hand washing | Peak flow meter/NM |
| Cefai C/1990 | Use of disposable ventilator tubing | Reusable ventilator tubing and humidifier/NM |
| Contant/1990 | Ethylene oxide sterilization of probes or discard after use | In-line temperature and oxygen monitor probes/NM |

**Table S2.** *Cont.*

| Authors/Year | Control Measures Directed Against the Source | Source/Predominant Involved Sites |
|---|---|---|
| Beck-Sague CM/1990 | Ethylene oxide terminal sterilization of pressure transducers between patients | Reusable pressure transducers in arterial lines/Blood |
| Ng PC/1989 | NM | IV nutrition fluids (presumed)/Blood |
| Kelkar R/1989 | Sterile disposable needles for methotrexate reconstitution | Multidose methotrexate and attached aspirating needle (presumed)/CSF |
| Hartstein AI/1988 | Ethylene oxide terminal sterilization of circuits and resuscitation bags, disposable gloves used for final packaging of sterilized circuits and bags | Reusable ventilator circuits and resuscitation bags/NM |
| Vandenbroucke-Grauls CM/1988 | Filters placed at end of inspiratory and expiratory tubing with technical modification of ventilator | Demand valve reservoir of ventilator/NM |
| Stone JW/1986 | Sterilization of resuscitator bags after use | Mouthpiece of resuscitator bag/NM |
| Sherertz RJ/1985 | Discard mattresses | Patient mattresses/Wound |
| Gervich DH/1985 | Removal of humidifiers | Bedside humidifiers/Blood |
| Kantor RJ/1983 | Discard diluted heparin after each shift | Heparinized saline solution/soft tissue |
| Irwin RS/1980 | Removal of all spirometers, sterile gloves for patient contact, strict hand washing | Ventilator spirometers/NM |
| Cunha BA/1980 | Restrict use of each respirometer to a single unit, volume measurements done at most distal portion of tubing, enforced and observed strict hand washing | Wright respirometers/NM |
| Reyes MP/1980 | Ethylene oxide sterilization of distilled water catheters, no distilled water to rinse catheters | Hospital-prepared distilled water/soft tissue |
| Lowes JA/1980 | Discontinuing use of malfunctioning bedpan washer, 1% hypochlorite disinfection of washed jugs followed by drainage | Bedpan and urine jugs/Urine |
| Abrutyn E/1978 | Autoclaving the baths, disinfection of heating elements after each use, drying of dialysis fluid bottles after removal from bath | Warming bath water/Dialysis drainage fliud |
| Smith PW/1977 | Removal of humidifiers | Bedside humidifiers/Blood |

**Table S2.** *Cont.*

| **Authors/Year** | **Control Measures Directed Against the Source** | **Source/Predominant Involved Sites** |
|---|---|---|
| | Outbreak (or Clusters) without an identified common source | |
| Authors/Year | Control measures directed against the source ( initial/advanced measures) | Source |
| Liu WL/2014 | preparation of disinfectants to adequately achieve environmental disinfection | NICS |
| Chen CH/2013 | NM/environmental disinfection | NICS |
| Apisarnthanarak A/2012 | NM/Environmental cleaning, contact isolation for patients, hand hygiene | NICS |
| Chmielarczyk A/2012 | NM/strict isolation, education of staff, hand hygiene and surface decontamination by vaporized hydrogen peroxide | NICS |
| Hosoglu S/2012 | NM/environmental disinfection | NICS |
| Lin WR/2011 | NM/Environmental cleaning, contact isolation for patients, hand hygiene | NICS |
| Choi WS/2010 | NM/Environmental cleaning, contact isolation for patients, hand hygiene | NICS |
| La Forgia C/2010 | NM/Environmental cleaning, contact isolation for patients, hand hygiene | NICS |
| Ray A/2010 | NM/strict isolation, education of staff, hand hygiene and surface decontamination by vaporized hydrogen peroxide | NICS |
| Chan PC/2007 | NM/contact isolation, reinforcing hand hygiene adherence, cohorting of nurses, and environmental cleaning | NICS |
| Wybo I/2007 | NM/contact precautions , hand hygiene , adherence, cohorting of nurses, and environmental cleaning , active surveillance cultures , controlled prescription and restricted use of broad-spectrum antibiotics | NICS |
| Pimentel JD/2007 | NM/Cleaning and environmental decontamination as well as staff education | NICS |
| El Shafie SS/2004 | NM/hand hygiene practices, extensive environmental cleaning, a closed suctioning system, education and review of other infection-control practices | NICS |
| Wang SH/2003 | NM/use of strict cohort nursing, hand hygiene environmental cleaning, and replacement of a dysfunctional high-efficiency particulate air filter | NICS |

**Table S2.** *Cont.*

| Authors/Year | Control Measures Directed Against the Source | Source/Predominant Involved Sites |
|---|---|---|
| Aygün G/2002 | NM/Unit closure, barrier isolation, environmental disinfection | NICS |
| Simor AE/2002 | NM/hand hygiene, strict patient isolation, meticulous environmental cleaning, and temporary closure of the unit | NICS |
| Fierobe L/2001 | NM/reinforcement of barrier safety measures, limitation of the number of admissions, and thorough environmental | NICS |
| D'Agata EM/2000 | NM/Contact isolation, surveillance cultures | NICS |
| Corbella X/2000 | NM/Unit closure, barrier isolation, environmental disinfection, controlled antibiotic use (carbapenem) | NICS |
| Biendo M/1999 | Cohorting and contact isolation for patients with active infection, chlorhexidine or iodine (4%) hand antisepsis/Unit closure and thorough environmental disinfection | NICS |
| Cox MT/1998 | NM/Environmental disinfection, disposable ventilator circuits, improved hand washing and changing of gloves between patient | NICS |
| Marques MB/1997 | Contact isolation/Education, monitoring, strict adherence to contact isolation | NICS |
| Riley TV/1996 | Body substance isolation, alcohol chlorhexidine hand disinfection, cohort patients, controlled antibiotic use (gentamicin and third-generation cephalosporins) (These measures were initially successful but then there was failure of control.) | NICS |
| Scerpella EG/1995 | NM/Cohort patients, cohort staff, contact isolation, phenol environmental disinfection, antibiotic use consultation | NICS |
| Crowe M/1995 | NM/Unit closure, cohort patients, cohort staff, contact isolation, phenol environmental disinfection, alcohol chlorhexidine hand disinfection or alcohol hand rub | NICS |
| Tankovic J/1994 | Unit closure, complete cleaning and disinfection, no transfer of known affected patients into unit | NICS |
| Go ES/1994 | NM/Cohort patients, controlled antibiotic use (imipenem), monitor hand washing and glove changes, wound irrigation with polymyxin B | NICS |

**Table S2.** *Cont.*

| Authors/Year | Control Measures Directed Against the Source | Source/Predominant Involved Sites |
|---|---|---|
| Struelens MJ/1993 | NM Contact isolation, alcohol hand antisepsis, sterilization of reusable ventilator tubing | NICS |
| Patterson J/1991 | NM/Cohort patients, appropriate glove changing and hand washing between patients | NICS |
| Buisson Y/1990 | NM/Controlled antibiotic use (amikacin), isolation of patients, hand washing | NICS |
| Allen KD/1987 | Cohort patients alcoholic chlor-hexidine hand antisepsis/Unit closure, environmental disinfection, discharge all colonized patients | NICS |
| Carlquist JF/1982 | NM/Strict isolation, antibacterial hand cleanser, environmental disinfection | NICS |
| Castle M/1978 | NM/Cohort patients, alcohol swab disinfection of Wright respirometer after each use, decrease routine saline irrigation for tracheobronchial suction, use small rubber-stoppered vials of saline for irrigation and discard after 24 hours, discard irrigating syringe after each use | NICS |

Abbreviations: aICU: adult intensive care units; CSF: Cerebrospinal fluid; mo: month; NICS: no identified common source; nICU: neonatal intensive care units; nm: no mention; sICU: surgical intensive care units.

**Table S3.** Infection prevention measures at Changhua Christian Hospital.

| Hand Hygiene Champions and Antibiotics Control Program | Contents |
|---|---|
| Contact isolation | 1. Implementation of enhanced contact isolation precautions (*i.e.*, strict adherence to hand hygiene before and after patient care and donning of clean gowns and gloves before patient care),<br>2. In addition, contact precautions and isolation were applied to every patient with a history of MDRAB colonization and infection who was re-admitted to CCH |
| Cohort | Placing MDRAB patients in single rooms or cohorting patients in open-structured units |
| Environmental cleaning | 1. Twice-daily environmental cleaning with a phenolic or quaternary ammonium disinfectant-detergent, as appropriate<br>2. A strict environmental cleaning policy was implemented according to CDC recommendations *[1] for rooms and for any objects that potentially contacted colonized patients. Certain devices (e.g., sphygmomanometers and stethoscopes) were dedicated for use on source patients whenever possible and were retained inside the room |

**Table S3.** *Cont.*

| **Hand Hygiene Champions and Antibiotics Control Program** | **Contents** |
| --- | --- |
| Meeting | 1. Regular weekly meetings were held with the staff (including physicians, nurses, physical therapists, and students) in affected areas.<br>2. In addition, the staff was periodically informed about the evolution of disease rates as part of the educational program |
| Strategy One: Systemic change for Antibiotics Control Program | 1. The infection control team (ICT) in CCH comprised an infection-control doctor, a pharmacist, an infection-control nurse and a microbiological technologist, and this team was extensively involved in the implementation of antimicrobial stewardship for all inpatients receiving antibiotic injections<br>2. The roles of the ICT included a review of antimicrobial orders with respect to the usage, dose, isolated pathogens and site of infection for all inpatients receiving parenteral antibiotics as well as consultation with the physicians before prescription of antibiotics.<br>3. A review was performed when antibiotic injections were prescribed.<br>4. Patients receiving carbapenem or anti-MDRAB agents were reviewed every week to facilitate therapy de-escalation.<br>5. When inappropriate antibiotic use was observed, the ICT members contacted the prescribing physicians. |
| Strategy Two: Education and training for Antibiotics Control Program | 1. Held education course every year to staffs for antimicrobial therapy such as the selection of antibiotics, dosage, treatment duration, and 3-day rule as well as to highlight examples of inappropriate antibiotic use |
| Strategy Three: Assessment and Feedback for Antibiotics Control Program | 1. To establish hand evaluation mechanism for antimicrobial administration , and regular audits<br>2. The duration of antimicrobial administration was limited to 2 weeks, and the ICT members included a cautionary message in the electronic medical chart. And, prolonged use of antibiotic injections over 2 weeks was not regarded as inappropriate for patients with infective septic arthritis, endocarditis, lung abscess and osteomyelitis. The duration was independently evaluated by the infection-control doctor and the clinical pharmacist.<br>3. When the message cautioning against continued antibiotic use was not heeded, the ICT members asked the prescriber to stop or change the antibiotics.<br>4. The appropriateness of antimicrobial use was decided according to the Sanford guide to antimicrobial therapy [*2]. Appropriateness of the duration was also evaluated according to the Sanford guide to antimicrobial therapy.<br>5. To feedback the results to the colleague<br>6. To hold team STEPPS meetings to strength team cohesion and teach communication skills |

**Table S3.** *Cont.*

| **Hand Hygiene Champions and Antibiotics Control Program** | **Contents** |
|---|---|
| Strategy Four: Workplace marked for Antibiotics Control Program | 1. Set computer screen saver and desktop to remind appropriate antimicrobial administration |
| Strategy Five: Creating a safety culture ethos hospital for Antibiotics Control Program | 1. Encourage patients to participate, and teach patients to remind staff for inappropriate antimicrobial adverse effect before antimicrobial prescription |
| Strategy Two: Education and training for Hand Hygiene Champions | 1. Hold education course for handle hand hygiene with a total of 140 sessions, and the total number of 8775 participates<br>2. Design hand-washing device to teach hand-washing techniques. |
| Strategy Four: Workplace marked for Hand Hygiene Champions | 1. Set computer screen saver and desktop to remind to wash hands<br>2. Executive head for hands washing activity |
| Strategy Three: Assessment and Feedback for Hand Hygiene Champions | 1. To establish hand hygiene evaluation mechanism, regular audits, and build compliance monitoring platform<br>2. To feedback the results to the colleague<br>3. To hold team STEPPS meetings to strength team cohesion and teach communication skills |
| Strategy Four: Workplace marked for Hand Hygiene Champions | 1. Set computer screen saver and desktop to remind wash hands<br>2. Executive leadership for hands washing activity |
| Strategy Five: Creating a safety culture ethos hospital for Hand Hygiene Champions | 1. Design Vital hand-wash routine<br>2. Encourage patients to participate, and teach patients to remind staff for washing their hands before patients care |

Notes: *[1] Centers for Disease Control and Prevention. Guidelines for environmental infection control in health-care facilities: recommendations of CDC and the Healthcare Infection Control Practices Advisory Committee (HICPAC) MMWR. 2003; 52(RR10):1–48; *[2] Gilbert DN, Moellering RC, Eliopoulos GM, *et al*. The Sanford Guide to Antimicrobial Therapy. 39th edn. Vermont: Antimicrobial Therapy; 2009.

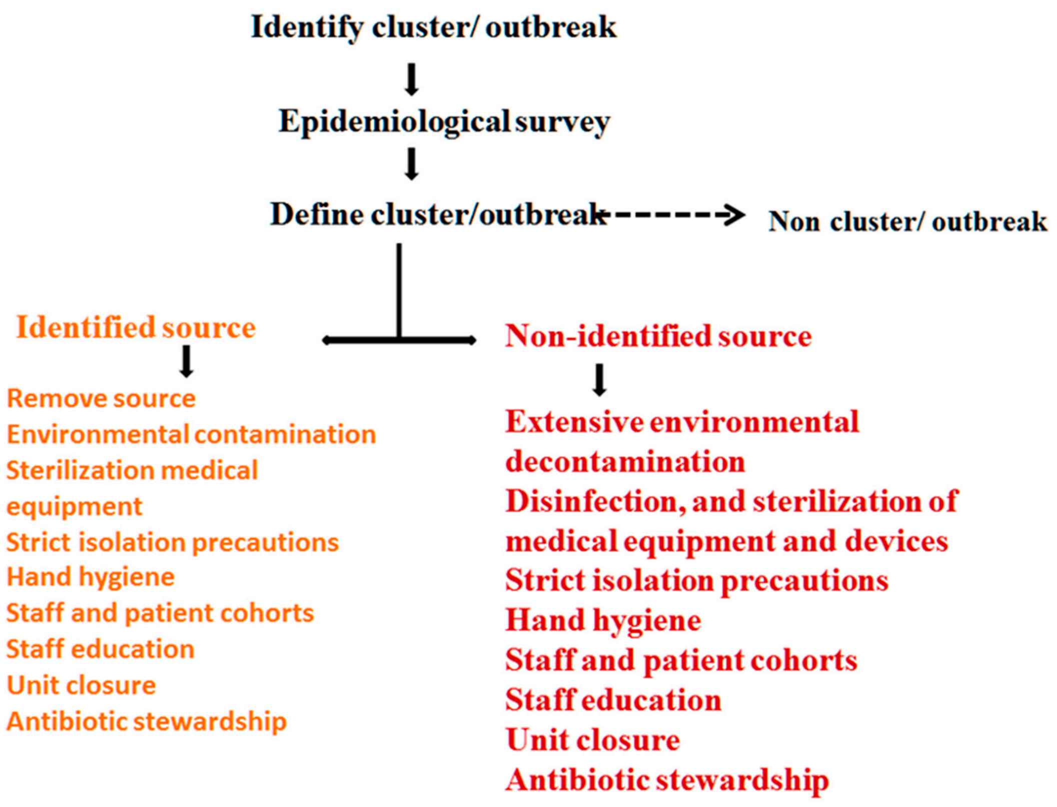


**Figure S1.** Process of cluster/outbreak at Changhua Christian Hospital.

© 2015 by the authors; licensee MDPI, Basel, Switzerland. This article is an open access article distributed under the terms and conditions of the Creative Commons Attribution license (http://creativecommons.org/licenses/by/4.0/).
